# Thermosensitive visible-light-excited visible-/NIR-luminescent complexes with lanthanide sensitized by the π-electronic system through intramolecular H-bonding

**DOI:** 10.3389/fchem.2022.1047960

**Published:** 2022-12-07

**Authors:** Hitomi Ohmagari, Nicolas Marets, Jun Kamata, Mayo Yoneyama, Takumi Miyauchi, Yuta Takahashi, Yukina Yamamoto, Yuto Ogihara, Daisuke Saito, Kenta Goto, Ayumi Ishii, Masako Kato, Miki Hasegawa

**Affiliations:** ^1^ Department of Chemistry and Biological Science, Aoyama Gakuin University, Sagamihara, Japan; ^2^ Mirai Molecular Materials Design Institute, Aoyama Gakuin University, Sagamihara, Japan; ^3^ Department of Chemistry, Faculty of Science, Hokkaido University, Sapporo, Japan; ^4^ Department of Applied Chemistry for Environment, School of Biological and Environmental Sciences, Kwansei Gakuin University, Sanda, Japan; ^5^ Institute for Materials Chemistry and Engineering, Kyushu University, Fukuoka, Japan; ^6^ Department of Natural and Environmental Science, Teikyo University of Science, Yamanashi, Japan

**Keywords:** lanthanide complexes, visible-light excitation, visible-/NIR-luminescence, intramolecular H-bonding, thermosensitive luminescence

## Abstract

Visible-luminescent lanthanide (Ln**L**) complexes with a highly planar tetradentate ligand were successfully developed for a visible-light solid-state excitation system. **L** was designed by using two 2-hydroxy-3-(2-pyridinyl)-benzaldehyde molecules bridged by ethylenediamine, which was then coordinated to a series of Ln ions (Ln = Nd, Sm, Eu, Gd, Tb, Dy, and Yb). From the measurement of single-crystal X-ray analysis of Eu**L**, two phenolic O atoms and two imine N atoms in **L** were coordinated to the Eu ion, and each π-electronic system took coplanar with the edged-pyridine moiety through an intramolecular hydrogen bond. The enol group on the phenolic skeleton changed to the keto form, and the pyridine was protonated. Thus, intramolecular proton transfer occurred in **L** after the complexation. Other complexes take isostructure. The space group is *P*-1, and the *c*-axis shrinks with decreasing temperature without a phase transition in Eu**L**. The yellow color caused by the planar structure of **L** can sensitize ff emission by visible light, and the luminescence color of each complex depends on central Ln ions. Furthermore, a phosphorescence band also appeared at rt with ff emission in Ln**L**. Drastic temperature dependence of luminescence was clarified quantitatively.

## Graphical Abstract

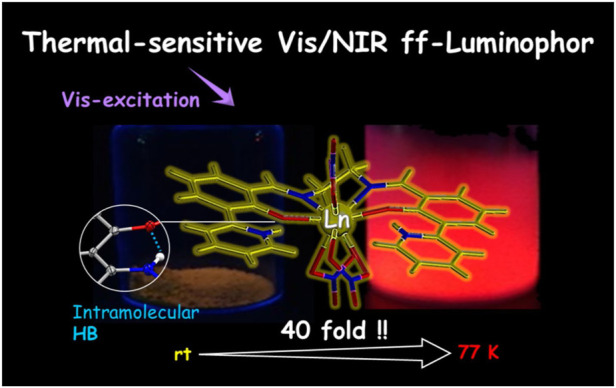

## 1 Introduction

**SCHEME 1:**
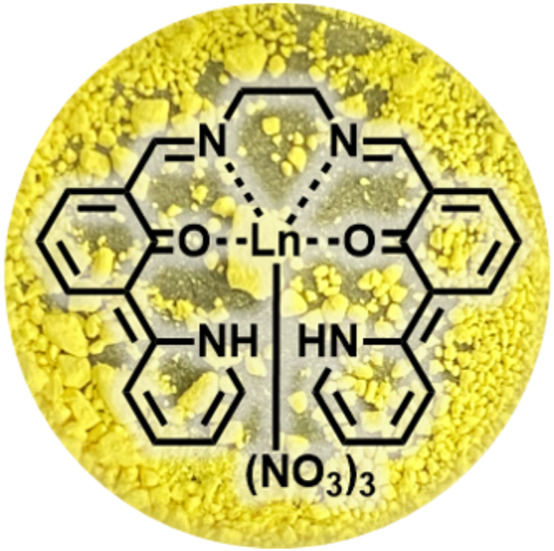
Molecular structure of Ln**L** and photograph of its powder (Eu). Every complex within the series of Ln complexes exhibits the same color.

**SCHEME 2:**
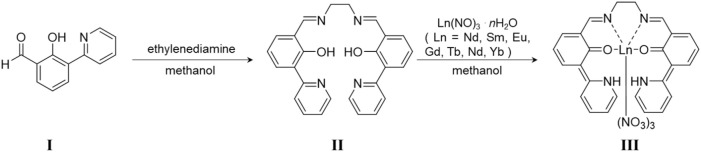
Preparation method of Ln**L**. **I** was synthesized following Ref. 23. **II** is ligand **L**, and **III** is Ln**L**.

Visible-light-excitable chromophores with visible-/NIR-light emission are very useful in physiological fields, for example, as biosensors, since visible light can prevent damage to cells and the luminescence of colored cells can be detected easily because the transparency of the skin is improved (Bender et al., 2002; Deiters et al., 2009; Jiang et al., 2010; Hasegawa et al., 2014; Ning et al., 2019; Parker et al., 2021). For the luminescence and structure stability in solutions and solid state, metal complexes considering chelate effect have potential. We designed and newly synthesized a tetradentate ligand (**L**) for a series of visible-light-excitable lanthanide (Ln) complexes with visible-/NIR-luminescence in the solid state (Schemes 1, 2). Ln ions in complexes exhibit characteristic band positions and have relatively long luminescence lifetimes in milliseconds because of their electron configuration. This is related to the parity selection rules (ff transitions), and the narrowness of these transitions is due to the shielding effects (electronic configuration). Therefore, their luminescence has the advantage of being easily distinguishable from other luminescent molecules in the living body. Based on these unique properties, the complex Ln**L** will be a candidate for applications to biological sensing materials. The ligand maintains its complexation with Ln by a chelate effect (Francis et al., 2020; Hasegawa and Ohmagari, 2020).

The luminescence color of Ln complexes is derived from Ln^3+^ species, for example, a Eu^3+^ complex shows pure red emission. These unique optical properties are due to the electronic configuration or electronic state for the *f*-orbital of the Ln ion. The band position of ff emissions for trivalent Ln ions depends on the number of f-electrons whose energy levels have been decided by the split under some rules such as relative magnitude of the interelectronic repulsion, spin–orbital coupling, and crystal-field effects (Binnemans, 2015).

In the case of Ln ions, the ff electron transitions are not involved in the reference vibration due to the shielding effect of the 5*s* and 5*p* orbitals in the outer shell. Thus, the usual method is to bind aromatic organic molecules with high absorption coefficients as ligands and promote ff emission of Ln through an intramolecular energy transfer *via* photo-excitation. This ff emission sensitization mechanism is known as the photo-antenna effect. However, a suitable excitation wavelength for the energy acceptor level for Ln^3+^, which shows emission in the visible light region, is mostly in the UV region, and there are very few reports on Ln complexes showing visible-light excitation and visible-light luminescence (Werts et al., 1999; Yang et al., 2004; Deun et al., 2005; Zhang et al., 2005; An et al., 2008; Chen et al., 2008; Andreiadis et al., 2009; He et al., 2009; Divya et al., 2010; He et al., 2010; Gu and Yan, 2013; Zhang et al., 2013; Sun et al., 2014; Xu et al., 2014; Aquino et al., 2021; Zhang et al., 2021).

The Eu^3+^ complex with 9-hydroxyphenal-1-one molecules (Deun et al., 2005) forms a highly coplanar π-electronic system, and its absorption band appears in the visible region. Similarly, a series of Eu^3+^ complexes with carbazole (He et al., 2009; He et al., 2010), tetrazole (Andreiadis et al., 2009), and *β*-diketone (Werts et al., 1999; Divya et al., 2010) also show visible-excitable luminescence due to coplanar π-electronic systems. There are few specific examples for the energy relaxation process in which energy transfer to the center metal occurs *via* not the triplet energy level but a singlet one (Yang et al., 2004; Kasprzycka et al., 2017; Aquino et al., 2021). In particular, the Eu^3+^ complex with a 2,4-di (2-pyridyl)-1,3,5-triazine derivative shows high luminescence quantum yield (QY; *φ*_L_) and *φ*_L_ = 0.52 (λ_ex_ = 402 nm) at room temperature (abbreviated to rt). Visible-light-excited luminescent Ln–Ln’ complexes based on ff absorption were reported in 1993 by Bünzli et al. (1993). They synthesized dinuclear Eu/Tb–Ln (Ln = Nd, Ho, and Yb) complexes with *p*-*tert*-butylcalix [8] arene and observed red emission in Eu–Ln excited at the absorption band position of the ^7^F_0_ →^5^D_0_ transition of Eu^3+^ (Bünzli et al., 1993).

In this investigation, we aim to clarify the solid state luminescence properties of a series of Ln**L** (Ln = Nd, Sm, Eu, Gd, Tb, Dy, and Yb) complexes by luminescence QY and lifetime measurement and with a single-crystal X-ray structure analysis and temperature-dependence of synchrotron X-ray diffraction (XRD) patterns for their sophisticated molecular and packing structures.

## 2 Results and discussion

### 2.1 Single-crystal structural analyses of Ln**L**

**FIGURE 1:**
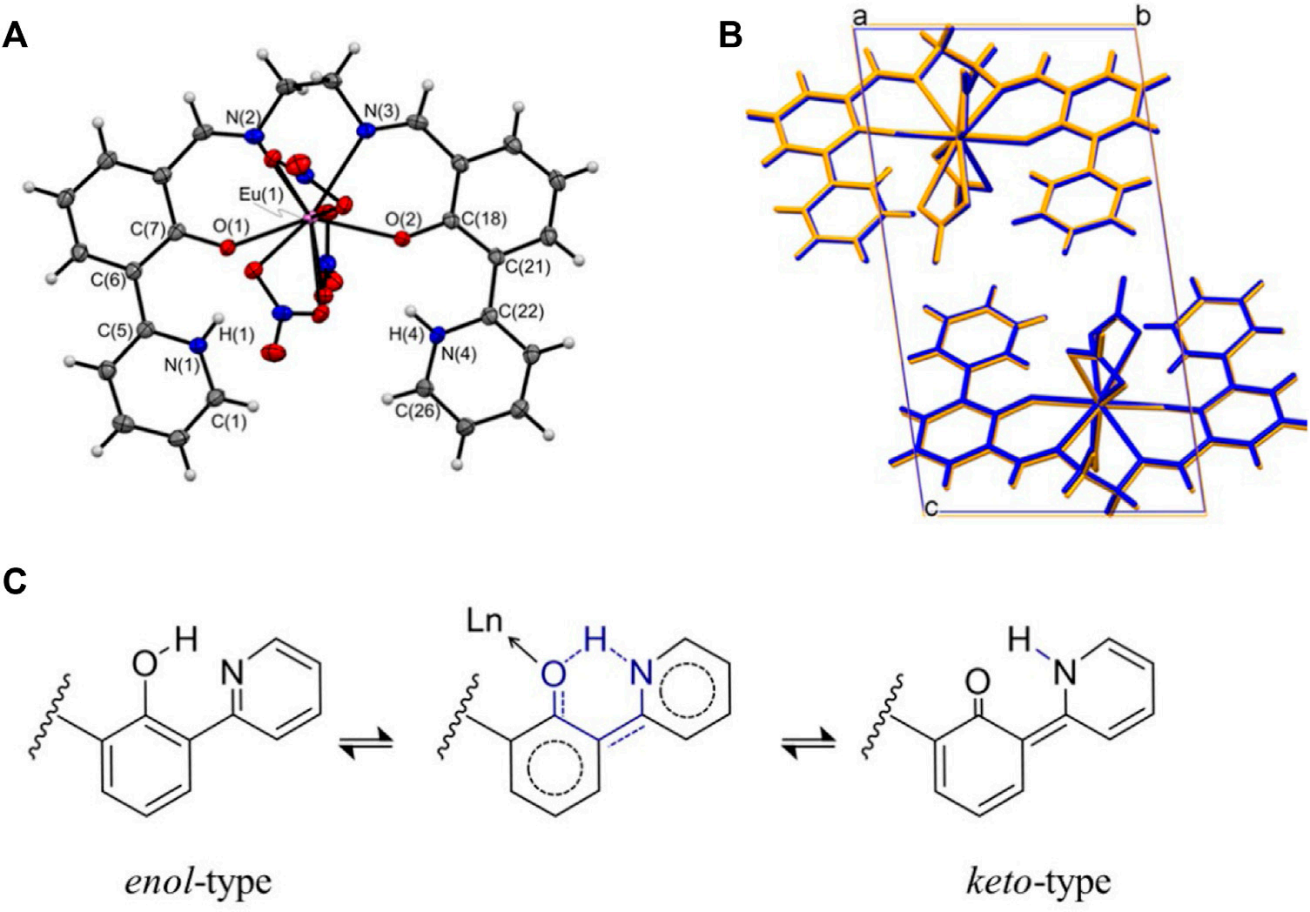
**(A)** ORTEP drawing of Eu**L** and **(B)** packing structures at 90 K (blue) and 300 K (orange) projected along the *a-*axis. **(C)** Possible *enol*–*keto* tautomerism and hydrogen-bonding in Ln**L**.

A series of Ln**L** complexes were obtained as yellowish crystals, and single-crystal X-ray structure diffraction analysis was performed to determine the molecular structures. In the case of Eu**L**, the, molecular structure and an overlay figure showing the packing structure at 90 and 300 K are shown in Figure 1. The crystal data and bond distances are summarized in Supplementary Tables S1, S3. **L** equatorially coordinates to Eu^3+^ as a tetradentate ligand at ONNO, and three nitrate ions as bidentate bind axially to Eu^3+^. Focusing on the 2-(pyridine-2-yl)phenol framework in **L**, intramolecular proton transformation (IPT) occurred before and after complexation, and **L** formed an intermediate form between keto and enol (Figure 1C). Actually, the bond lengths for C(7)-O(1) and C(18)-O(2) for the phenol moiety in Ln**L** are almost 1.31 Å, which is the shorter- and longer-value than actual values of 1.38–1.39 Å for C-OH and 1.22 Å for C=O, respectively, while the angle C-N-C of the pyridine skeleton of Eu**L** corresponds to that of a normal pyridine rather than piperidine. Thus, the proton will locate between O of phenol and N of pyridine in the complex and contribute to keep high planarity of **L**. Moreover, three nitrate ions coordinated to a Eu^3+^ ion to balance the charge, which confirms that Ln**L** is in the keto–amine form with the coordination number 10. The molecular structures and potential energies for IPT with keto–enol tautomerism in similar molecules with **L** have been experimentally (LeGourriérec et al., 1998; Hasegawa et al., 1999; Basarić and Wan, 2006) and theoretically (Korth et al., 2002; Li et al., 2017; Wang et al., 2019) demonstrated.

**FIGURE 2:**
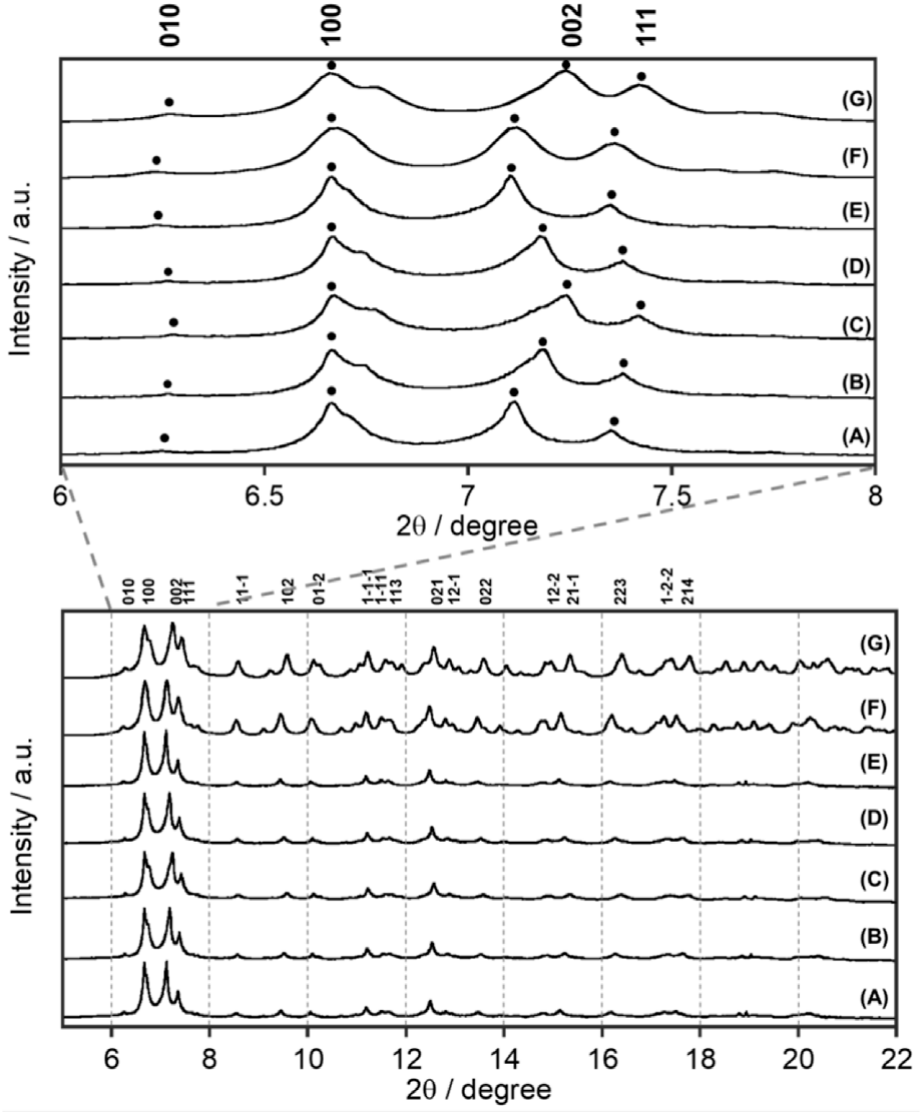
Temperature dependence of synchrotron XRD patterns of Eu**L** at X-ray wavelength λ = 0.998983 Å: **(A)** 300 K, **(B)** 200 K, **(C)** 90 K, **(D)** 200 K, and **(E)** 300 K. Simulated pattern from single-crystal X-ray analyses (Figure 1) at **(F)** 300 K and **(G)** 100 K.

The crystal system of Eu**L** is *triclinic* with a space group of *P*-1. Two molecules form a unit cell (*Z* = 2). These features are consistent with the 100 K data. In contrast, when viewed from the *a*- and *b*-axes, the positions of the atoms in the unit cell are shifted significantly in the *c*-axis direction at 300 K. The length of the unit cell along the *c*-axis is 16.41 Å at 300 K, which is almost 0.30 Å higher than that at 100 K. The unit cell lengths along the *a*- and *b*-axes are almost the same regardless of temperature, whereas the temperature-dependent stretching along the *c*-axis is four times larger than that along the *b*-axis and 40-fold greater than that along the *a*-axis. This trend supports the results of temperature-dependent results of synchrotron XRD measurements (Figure 2). Single crystals of Gd**L** and Tb**L** were prepared and subjected to single-crystal X-ray structural analysis. The results are similar to those for Eu**L**, i.e., the crystal system is *triclinic*, the space group is *P*-1, and the *Z* value is 2. The ligand is tetradentate to the central metal, and three nitrate ions are coordinated to the metal. The molecular and packing structures for Tb**L** and Gd**L** are shown in Supplementary Figures S1 and Supplementary Figures S2, respectively, and the crystal data and bond lengths are shown in Supplementary Table S2. The crystal data and bond lengths for Ln**L** are summarized in Supplementary Table S3. Interestingly, there are two differences between Tb**L**, Eu**L**, and Gd**L**. First, from the side view of molecules in Supplementary Figure S1, it can be seen that the pyridine ring on one side of Tb**L** is more distorted than those for Eu**L** and Gd**L**. The second difference is in the coordination mode of the nitrate ions. In Eu**L** and Gd**L**, the three nitrate ions are coordinated in a bidentate fashion, whereas in Tb**L**, one of the three nitrate ions is monodentate. In fact, the distance Ln-O (7) between the oxygen atom of the nitrate ion and Tb ion is 3.3 Å, but it is 2.5 Å in the case of Eu**L** and Gd**L** with a difference of about 0.8 Å.

Nd**L**, Sm**L**, Dy**L**, and Yb**L** were also crystallized, and single-crystal X-ray structure analyses were performed (Supplementary Figures S3–S6 and Supplementary Table S4). The space group of these complexes is also *P*-1. π-electronic systems in Tb**L**, Dy**L**, and Yb**L** distorted resulting in monodentate NO_3_^−^ coordination, and these crystals contain DMF. Their structural properties correspond to that of Tb**L**. Table 1 summarizes the dihedral angles localized on right and left wings, the dihedral angle between right and left wings (*ω*_*Left*_, *ω*_*Right*_, and *ξ*, respectively), and the average value of two hydrogen bond distance (*r*_HB_) and ionic radii (Cotton, 2006) for a series of Ln**L** complexes. *ω*_*Left*_ differs from *ω*_*Right*_, while every value of the right or left one is equal in Nd**L**, Sm**L**, Eu**L**, and Gd**L** and in Tb**L**, Dy**L**, and Yb**L**. Their molecular structure differences clearly enhanced to the unit cell information, for example, the *c*-axis drastically changes (Supplementary Table S4). It suggests that **L** coordinates without involving ionic radii of Ln, but ionic radii change the coordination number and structures. It was expected that there is some correlation between *r*_HB_ and the lanthanoid contraction, but no less clear differences have been observed.

**TABLE 1:** Comparison of dihedral angles and ionic radii of Ln**L** (Ln = Nd, Sm, Eu, Gd, Tb, Dy, and Yb). *ω*_*Left*_ and *ω*_*Right*_ are dihedral angles of aromatic rings crosslinked at C (6)–C (5) and C (21)–C (22), respectively. The ξ value is the dihedral angle between the two π-electronic systems. *r*_HB_ is the average value of two hydrogen bond distances.

	Nd**L**	Sm**L**	Eu**L**	Gd**L**	Tb**L**	Dy**L**	Yb**L**
Space group	*P*-1	*P*-1	*P*-1	*P*-1	*P*-1	*P*-1	*P*-1
*ω*_*Left*_/^o^	7.1	7.1	7.1	7.0	8.1	8.3	8.4
*ω*_*Right*_/^o^	3.9	4.2	4.3	4.4	2.4	2.5	2.5
*ξ*/^o^	0.96	0.63	0.54	0.68	1.70	1.83	1.90
*r*_HB_	1.75	1.80	1.81	1.80	1.79	1.81	1.82
Ionic radii/Å^[31]^	0.98	0.96	0.95	0.94	0.92	0.91	0.87

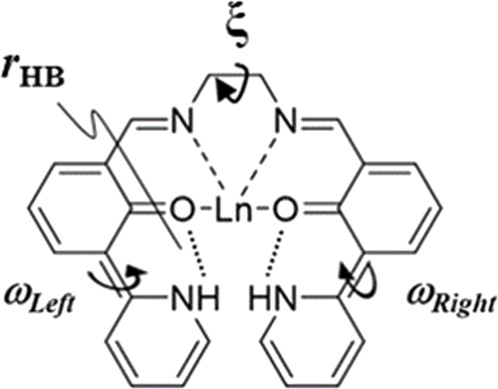

### 2.2 Synchrotron X-ray diffraction

Synchrotron XRD patterns of Eu**L** at various temperatures are shown in Figure 2, where the temperature was decreased from 300 to 90 K and then increased to 300 K again. Eu**L** shows a sharp diffraction pattern at all temperatures. Interestingly, when the *c*-axis of the crystal lattice is not involved, such as for the (010) and (100) planes, no temperature dependences were observed. These peaks associated with (002) and (111) planes, which have a *c*-axis component, shifted to a high angle and became broader at a low temperature. This indicates that the *c*-axis elongates with increasing temperature. From the observed results at 90 and 300 K in Supplementary Table S1, the difference of *c*-axis was 0.31 Å but those of *a-* and *b-*axes were negligibly small. In addition, the XRD pattern obtained by gradually decreasing the temperature from 300 K and then returning to 300 K reproduces the initial 300 K XRD pattern, indicating that the elongation along the *c*-axis is reversible with temperature.

### 2.3 Thermosensitive/visible-excited luminescence of Ln**L**

Electronic absorption spectra provide considerable information about the electronic state and molecular structure of a molecule. Supplementary Figure S7 shows electronic absorption spectra of ligand **L** and Eu**L**, Tb**L**, and Gd**L** complexes in acetonitrile. The ligands show broad bands around 335–430 nm assigned to the ππ* transitions localized on **L**. Upon complexation, the band at 335 nm is heavily red-shifted and appears around 380 nm. This is due to ππ* transitions of the ligand and is independent of the type of metal ion. Ln**L**, the mother skeleton, shows a ππ* absorption band around 315 nm; thus, the absorption band for Ln**L** is red-shifted by 65 nm. This shift is presumably due to the increased planarity of the electronic system *via* intra-ligand hydrogen bonding. In other words, the electronic state for the ligand is only realized after complexation. The latter band at 430 nm disappeared after the complexation, meaning that it would be an n-π* electronic transition localized on the ligand.

**FIGURE 3:**
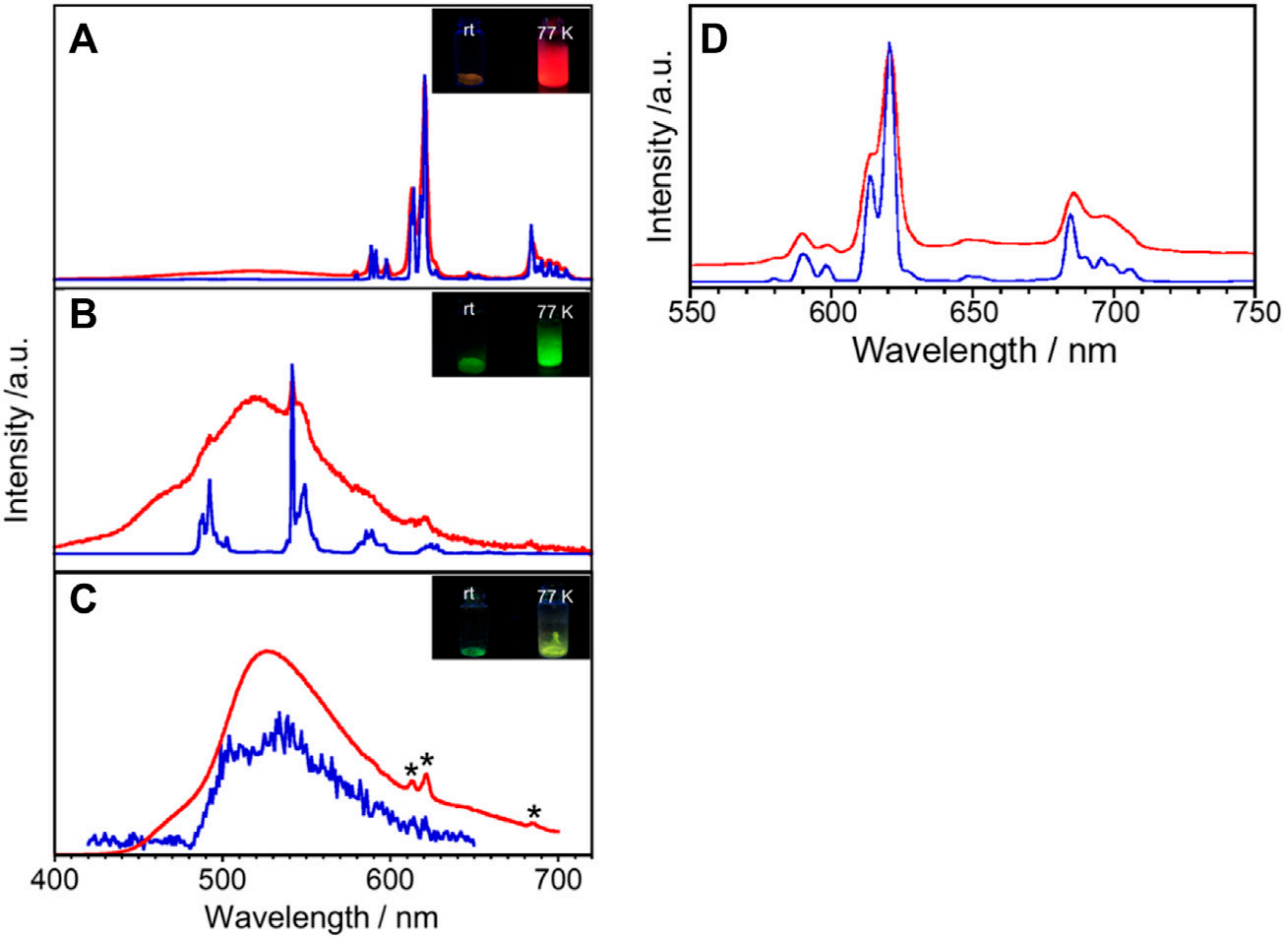
Temperature dependence of the solid-state luminescence spectra of **(A)** Eu**L**, **(B)** Tb**L**, and **(C)** Gd**L** at rt (red) and 77 K (blue). *λ*_ex_ = 380 nm, *due to impurity. Inset in **(A)**, **(B)**, and **(C)**: photograph of luminescence of Eu**L**, Tb**L**, and Gd**L**, respectively, at various temperatures. **(D)** Eu**L** also shows a luminescence band at *λ*_ex_ = 400 nm.

Generally, the Gd complex is used as a reference molecule to determine the electronic state for the energy donor electron system. For example, since multidentate ligands such as **L** take on various conformations in the metal-free state, Gd complexes are commonly used to determine fluorescence and phosphorescence when they have the same structure as ligands of Eu and Tb complexes. Figure 3C shows emission spectra of Gd**L** in the solid state at rt and 77 K. Gd**L** exhibits broad emission bands around 440–480 nm and 480–650 nm at rt. The former luminescence band relatively becomes weaker upon cooling and the latter one becomes stronger, which suggests that it is due to the fluorescence band of **L**. Luminescence lifetime is also suggested from the aforementioned aspect (Table 2, Supplementary Figure S8C,D). The *τ* value at *λ*_mon_ = 470 nm is estimated to two components: 0.10 (amp. 99.3%) and 1.32 (0.7%) ns at rt and 0.15 (98.0%) and 3.38 (2.0%) at 77 K. On the other hand, the value of *λ*_mon_ = 540 nm is estimated as two luminescent components at rt, 3.8 μs (99.9%) and 0.50 ms (0.1%), and three at 77 K, 0.05 ms (58.5%), 0.30 ms (39.5%), and 1.20 ms (2.0%). Thus, this band is phosphorescence of **L**. The *φ*_ff_ value for Gd**L** at rt was <0.1%; at 77 K, it increased to 44.0%. These results indicate that Gd**L** shows remarkable thermosensitive properties and will discuss the sensitization of the ff-luminescence behavior of other Ln**L** complexes in the following paragraphs. Furthermore, the appearance of the phosphorescence band at ambient temperature means that the heavy metal effect in Gd**L** is efficient.

**TABLE 2:** Luminescence QY and lifetimes of a series of Ln**L** complexes.

	Temp	*φ*_L_	λ_obs_/nm	λ_ex_/nm	λ_mon_/nm	*τ*_av_/ms	*τ*_1_/ms (amp.)	*τ*_2_/ms (amp	*τ*_3_/ms (amp.)
Eu**L**	rt	1.3%	550–750	365	621	0.23	6.2 × 10^−5^ (87.6%)	0.17 (11.2%)	0.45 (1.2%)
	77 K	57.3%	550–750			0.82	0.82 (100%)	-	-
Tb**L**	rt	n.d.	450–700	370	542				-
	77 K	41.6%	450–700			1.166	0.182 (33.4%)	0.649 (29.2%)	1.456 (37%)
Nd**L** ^a^	rt	0.02%	927–1,140	-	-	-	-	-	-
Sm**L** ^a^	rt	0.68%	800–1,500	-	-	-	-	-	-
Dy**L** ^a^	rt	n.d	-	-	-	-	-	-	-
Yb**L** ^a^	rt	0.23%	887–1,152	-	-	-	-	-	-

^a^ Luminescence QY at 77 K and lifetimes in the NIR wavelength region were not observed due to the apparatus.

The solid-state emission spectra of Eu**L** are shown in Figures 3A,D at *λ*_ex_ = 380 and 400 nm. The bands around 579, 591, 621, 647, and 696 nm at rt are assigned to the ^*5*^D_0_→^*7*^F_0_, ^*5*^D_0_→^*7*^F_1_, ^*5*^D_0_→^*7*^F_2_, ^*5*^D_0_→^*7*^F_3_, and ^*5*^D_0_→^*7*^F_4_ transitions of Eu^3+^, respectively. The corresponding emission bands at 77 K appear at almost the same wavelength position but become sharper than those at rt. The red emission is confirmed visually as shown in Figure 3A. The lowest excitation band position monitored at each luminescence band position well reproduces the absorption spectrum in acetonitrile (Supplementary Figure S7), indicating that the emission of Eu^3+^ is due to the energy transfer from the ligand. Additionally, Eu**L** exhibits a broad band at 450–575 nm, similar to the spectrum of Gd**L**. As described previously, the emission of Gd complexes is due to fluorescence and phosphorescence of the ligands. In other words, the broad band for Eu**L** is a ligand-derived emission band with metal-centered sharp bands. The solid-state emission and excitation spectra of Tb**L** are shown in Figure 3B and Supplementary Figure S9. The complex luminescence band around 493, 542, 590, and 628 nm at 77 K are assigned to the ^*5*^D_4_→^*7*^F_6_, ^*5*^D_4_→^*7*^F_5_, ^*5*^D_4_→^*7*^F_4_, and ^*5*^D_4_→^*7*^F_3_ transitions of Tb^3+^, respectively. It also exhibits a broad emission band at 520 nm at rt derived from the ligand, which imposed with a sharp emission band of Tb^3+^. Under the UV irradiation, Tb**L** shows a green emission at rt and 77 K as shown in Figure 3B, inset. However, the origin of the luminescence differs; the green luminescence at rt was ligand-dominated, while that was due to the ff transitions at 77 K.

Luminescence profiles, lifetimes, and QY for a series of Ln**L** complexes are shown in Supplementary Figure S8 and Table 2. The *φ*_L_ value for Eu**L** at 77 K (57.3%) is about 40 times higher than that at rt (1.3%). Such temperature-dependent Eu complexes have rarely been reported. Under the molecular design concept which having an absorption band at the visible wavelength region, it unexpectedly accelerates a drastic spectral change in Eu emission by temperature stimuli. The luminescence lifetime for Eu**L** at 621 nm at 77 K is 0.82 ms as a single component. On the other hand, at rt, the luminescence lifetime for Eu**L** is analyzed as three components, 6.2 × 10^–5^, 0.17, and 0.45 ms at 621 nm, meaning that various transitions overlap at this wavelength as described previously (Figure 3A). The *φ*_L_ value for Tb**L** at rt could not be estimated due to being negligibly weak. Remarkably, the *φ*_L_ value for Tb**L** at 77 K increases up to 46.1%. Two luminescence components for Tb**L** at rt were estimated as 0.091 and 0.661 ms monitored at 542 nm. From the comparison of luminescence lifetimes of Gd**L**, the shorter-lifetime components for Eu**L** and Tb**L** are assumed to be due to ππ* transitions in the ligand.

Quantitative energy relaxation can be discussed by using aforementioned luminescence properties of the Eu complex, since the electronic transitions of Eu^3+^ independently appear in their magnetic dipole transitions and electric transitions. The luminescence efficiency (Bünzli et al., 2007) of Eu^3+^ sensitized by the ligand (*φ*_L_ is determined by the triplet yield of the ligand (*φ*_ISC_)) (El-Sayed, 1963), the efficiency of the energy transfer (*η*_EnT_), and the efficiency of the metal-centered luminescence (*φ*_Ln_) are expressed as follows:

$$\varphi_{L}=\varphi_{ISC}\times \eta_{EnT}\times \varphi_{\mathrm{Ln}},$$ (1)

$$\varphi_{\mathrm{Ln}}=k_{R}\;/\;\left(k_{R}+k_{NR}\right)=k_{R}\times \tau_{av},$$ (2)

$$k_{R}=A_{MD,0}\times n^{3}\times \left(I_{tot}\;/\;I_{MD}\right).$$ (3)

The photophysical properties of Eu**L** are calculated using Eqs 1–3, where *φ*_ISC_ = 1 (Bünzli et al., 2007; Hasegawa et al., 2018) and *n* = 1.5 for the solid state and *k*_R_ and *k*_NR_ are radiative and non-radiative decay rates, respectively. *A*_MD,0_ is a constant relating to the spontaneous emission probability for the ^5^D_0_→^7^F_0_ of Eu^3+^ in vacuum (14.65 s^−1^). *I*_tot_ and *I*_MD_ are integrated luminescence intensity of the total luminescence of Eu**L** (550–750 nm) and the region of ^5^D_0_→^7^F_1_ (583–603 nm), respectively, and are determined from their spectral area recorded on an absolute emission quantum yield spectrometer C9920-02. The calculated results are shown in Table 3. It is worth noting that the *η*_EnT_ value shows obvious temperature dependence, for example, the values at rt and 77 K are 0.084% and ≈100%, respectively.

**TABLE 3:** Photophysical parameters of Eu**L** in the solid state. (^a^: λ_ex_ = 380 nm, λ_mon_ = 583–603 nm,^b^: λ_ex_ = 380 nm, and λ_mon_ = 550–750 nm).

	Temp	*I*_MD_^a^	*I*_tot_^b^	*τ*_av_/s	*k*_R_/s^−1^	*k*_NR_/s^−1^	*φ*_Ln_	*η*_EnT_
Eu**L**	rt	9.7 × 10^−4^	1.3 × 10^−2^	2.3 × 10^−4^	662	3,685	0.15	0.085
	77 K	0.053	0.57	8.2 × 10^−4^	531	687	0.43	≈1.0

**FIGURE 4:**
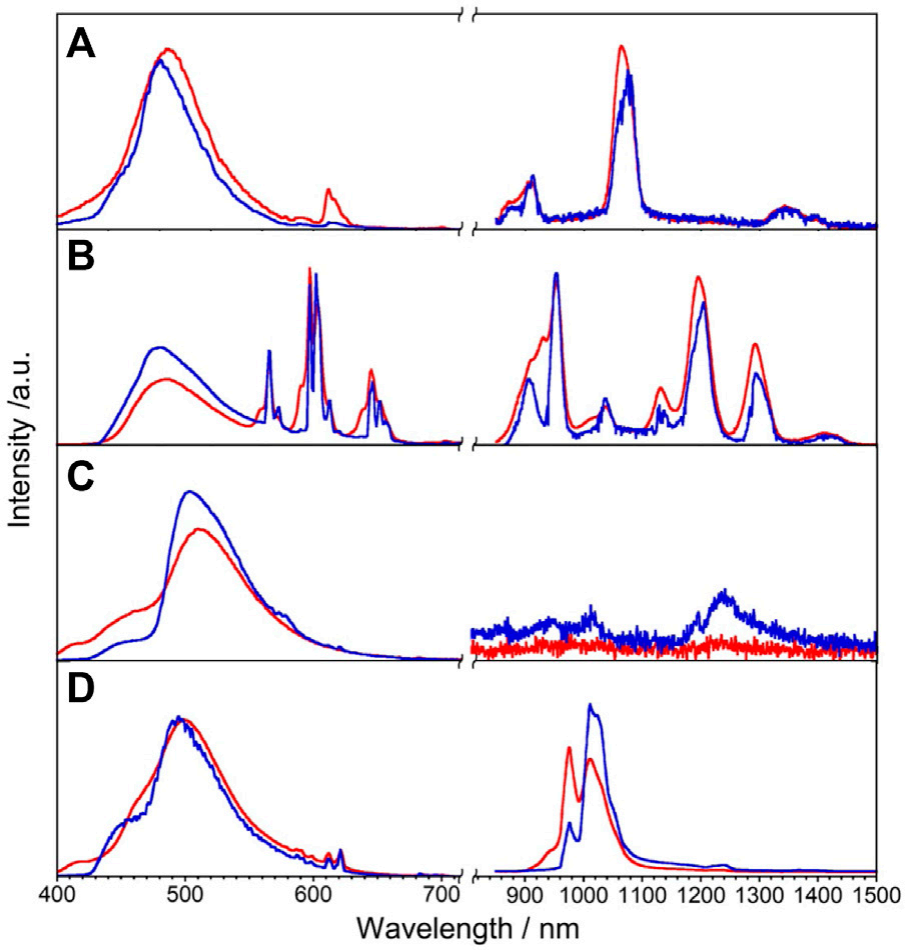
Luminescence spectra of Ln**L**, Ln = Nd **(A)**, Sm **(B)**, Dy **(C)**, and Yb **(D)**, in the solid state at rt (red) and 77 K (blue), *λ*_ex_ = 380 nm.

We expected that the energy donor level of **L** will also be efficient to sensitize NIR luminescence of Nd, Sm, Dy, and Yb *via* the intramolecular energy transfer. The luminescence spectra of Ln**L** (Ln = Nd, Sm, Dy, and Yb) at rt and 77 K are shown in Figure 4. Nd**L** clearly shows emission bands around 911, 1,063, and 1,340 nm at rt, which are assigned to ^4^F_3/2_ → ^4^I_*J*_ (*J* = 9/2, 11/2, and 13/2, respectively) transitions of Nd^3+^. The band positions and relative intensities are stable at 77 K. The broad band at a similar position observed at 420 nm of Gd**L** also appeared in the case of Nd**L** as ππ* transitions. Sm**L** shows emission bands around 951, 1,037, 1,132, 1,191, and 1,292 nm assigned to the ^4^G_5/2_→^6^F_*J*_ (*J* = 5/2, 7/2, 9/2, 11/2, and 13/2, respectively) transitions of Sm^3+^, accompanied with ππ* luminescence bands. Dy**L** shows luminescence bands around 570 nm in the visible region and around 1,010–1,190 nm in the NIR region, attributed to the ^4^F_9/2_ → ^6^H_7/2_ and ^4^F_9/2_ →^6^H_5/2_ transitions, respectively. In general, Dy^3+^ shows emission bands in the wavelength region of visible-NIR (around 475, 570, 660, 750, 1,000, 1,180, 1,270, and 1,400 nm), but Dy**L** does not show these bands because of the superimposition between ππ* luminescence band and ff ones. Yb**L** shows the emission band at 970–1,080 nm assigned to the ^2^F_5/2_→^2^F_7/2_ transition of Yb^3+^. The split band shape of Yb**L** shows temperature dependence and becomes shaper at 77 K than at rt. The absolute luminescence quantum yields ff at rt in the NIR region for Nd**L**, Sm**L**, Dy**L**, and Yb**L** are 0.02%, 0.68%, <10^–6^%, and 0.23%, respectively.

**FIGURE 5:**
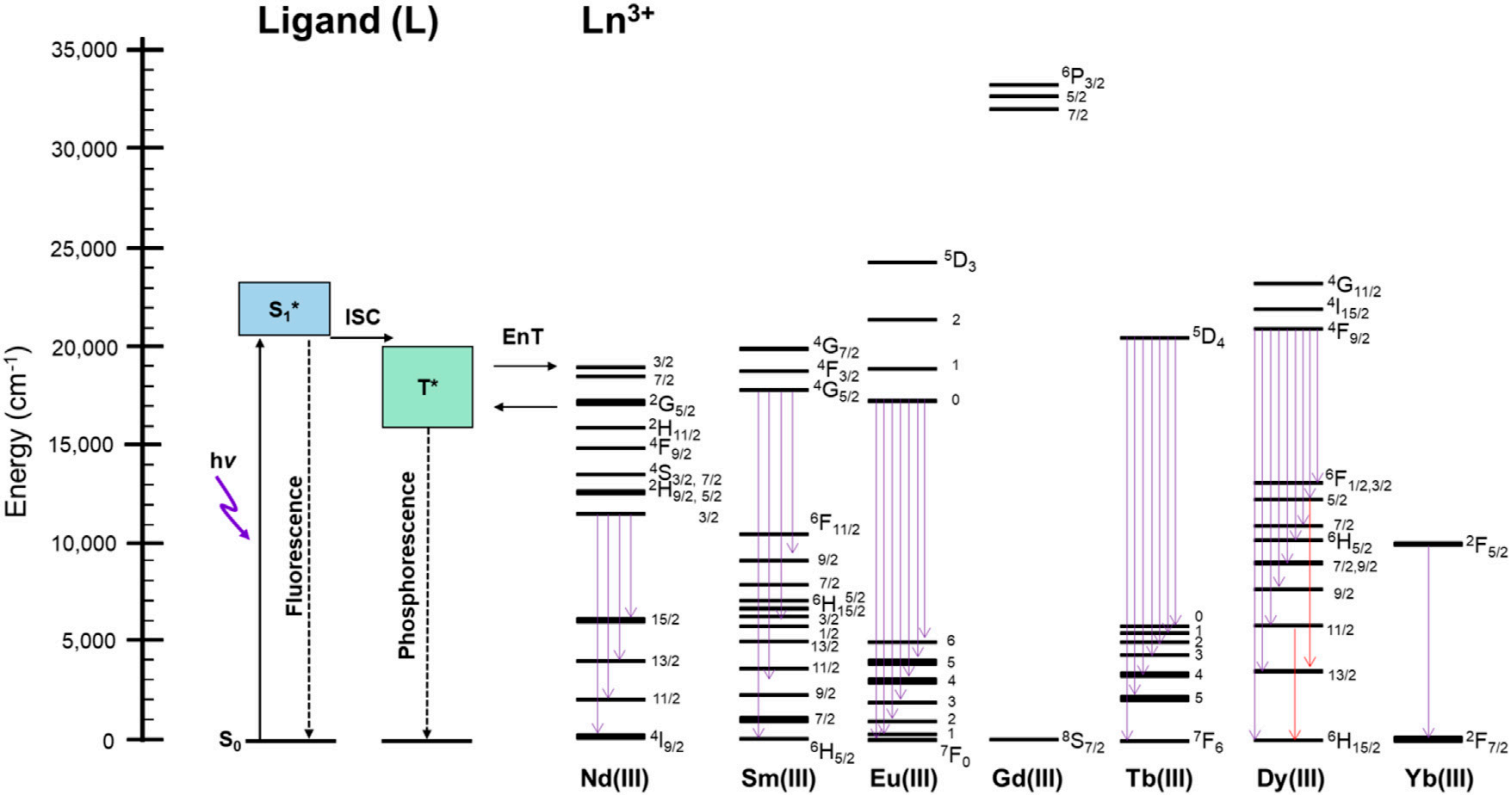
Schematic representation of the energy relaxation process at rt and 77 K of Ln**L** in the solid state. ISC: intersystem crossing; EnT: energy transfer; Flu.: fluorescence; Phos.: phosphorescence.

Figure 5 shows the schematic representation for the explanation of the thermosensitive energy relaxation for Ln**L**. These complexes take the photoexcited donor state by visible light. The fluorescence and phosphorescence bands of Gd**L** with peak position at 460–470 nm, respectively, appear mostly in the similar position with relatively large band width (Figure 3). Then, the exciton at T^*^ *via* intersystem crossing undergoes energy relaxation due to phosphorescence and transfers photoexcited energy to the metal. This is based on the fact that phosphorescence and ff emission are observed simultaneously. Interestingly, these complexes with Nd, Sm, Eu, Tb, Dy, and Yb also retain the ligand-centered ππ* luminescence at rt and 77 K with their ff emission. As described previously, the ligand emission at rt is more dominant in the case of Tb**L** than in the case of Eu**L** because the acceptor level for Tb^3+^ is higher in energy than the acceptor level for Eu^3+^, and then the ligand emission strongly appears more than ff emission of Tb^3+^ at rt and *vice versa* at 77 K. In particular, Eu**L** obviously exhibits the temperature dependences in luminescence spectra. The multiphonon mechanism is one of the possibilities to enhance such temperature dependence of ff emissions in Ln complexes (Kreidt et al., 2018). Actually, the stretching vibration of N–H was observed around 3,100 cm^−1^ in Eu**L** and Tb**L** originated from **L** from the measurement of FT-IR (ATR method) as shown in Supplementary Figure S10. The band position of Eu**L**, for example, at 621 nm (16,100 cm^−1^) corresponds to the five times of N-H vibration, which will contribute to the temperature dependence of Eu emission with **L**. Thus, the ligand **L** does not only sensitize the ff emission at visible light but also results in both ligand-/Ln-centered emission or their selectivity at various temperatures due to the energy levels of donor and acceptor levels.

## 3 Conclusion

Yellowish complexes, Ln**L** (Ln = Nd, Sm, Eu, Gd, Tb, Dy, and Yb), are sufficient electronic structures as visible-light-excited visible dual luminescence compounds taken into account the π-electronic system coplanarity through intramolecular hydrogen bonding. Molecular and packing structures for a series of Ln**L** complexes take isostructure. **L** coordinates to Ln^3+^ as a tetradentate ligand. The intramolecular hydrogen bonds through keto–amine and enol–imine isomerization keep the molecular planarity of the ligand moiety, which results that the electronic absorption bands appear at the visible region. Single-crystal X-ray structural analysis of the series of Ln complexes was also performed. A significant change in the crystal structure occurs after gadolinium. Synchrotron XRD and single-crystal X-ray structural analysis show temperature dependence in the *c*-axis direction, but no influence of the temperature dependence was observed in the excited state of **L**.

Tb**L** exhibits ff emission and ligand emission simultaneously with temperature dependence based on the thermal equilibrium between energy donor and acceptor levels. The absolute luminescence quantum yields for Eu**L** at 77 K are much higher than those at rt, and the intramolecular energy transfer was quantitatively discussed by their luminescence lifetimes. The QYs of ff emissions of Eu**L** also show drastic temperature dependence due to the multiphonon effect relating to the N–H vibration of **L**. The band positions of ππ* emission of Gd**L** support to explain the aforementioned phenomena from the viewpoint of the intramolecular energy transfer mechanism. The ligand **L** also acts as an energy donor to induce NIR luminescence of several kinds of Ln^3+^. It was found that these cases show metal selectivity to Dy and thermo-sensitivity. Thus, it is surely expected that a series of Ln**L** complexes are applicable to luminescent thermo-sensors, especially toward self-calibrated thermometers (Rocha et al., 2016), which have never used the excitation light in the visible region.

## 4 Materials and methods

### 4.1 Materials

The commercially available chemicals were of analytical reagent grade and were used without further purification: 3-bromosalicylaldehyde, tributyl (2-pyridyl)tin, tetrakis (triphenyl-phosphine)palladium (0), dry ethylenediamine, and Ln (NO_3_)·6H_2_O. All other starting materials were of analytical grade, obtained from commercial sources, and were used without further purification.

### 4.2 Synthesis of 6,6’-((1E,1′E)-(ethane-1,2-diylbis(azaneylyliden-e))bis(methaneylylidene))bis(2-(pyridine-2-yl)phenol) (II: **L**)

2-Hydroxy-3-(2-pyridinyl)-benzaldehyde(**I**) was synthesized through the process already reported in Honda and Arai (2016). Yield: 460 mg (47%); ^1^H-NMR (500 MHz, CDCl_3_) δ 10.62 (t, J = 14.9 Hz, 1H), 8.56 (td, J = 3.3, 1.7 Hz, 1H), 8.06 (dd, J = 7.4, 1.7 Hz, 1H), 7.97 (d, J = 8.0 Hz, 1H), 7.86–7.92 (m, 2H), 7.32–7.35 (m, 1H), and 7.00 (t, J = 7.7 Hz, 1H).

**L** was synthesized from two equivalents of (**I**) bridged with an ethylenediamine and obtained as a yellowish powder according to Hasegawa et al. (2014). Yield: 234 mg (52%); 1H-NMR (500 MHz, CDCl_3_) δ8.67 (d, J = 4.0 Hz, 2H), 8.53 (s, 2H), 8.05 (d, J = 8.0 Hz, 2H), 7.94 (d, J = 7.4 Hz, 2H), 7.74–7.77 (m, 2H), 7.23 (ddd, J = 7.4, 5.2, 1.1 Hz, 2H), 6.98 (t, J = 7.4 Hz, 2H), and 3.99 (t, J = 14.9 Hz, 4H).

### 4.3 Synthesis of Ln complexes with **L** (III: Ln**L**) (Eu**L**, Tb**L**, Gd**L**, Nd**L**, Sm**L**, Dy**L**, and Yb**L**)

**L** (50.2 mg, 0.118 mmol) was dissolved in 50 ml of methanol and stirred with a solution of Eu(NO_3_)_3_·6H_2_O (52.79 mg, 0.118 mmol) in 5 ml of methanol at rt for 3 h. The mixture was passed through a membrane filter, and Ln**L** was obtained as a yellowish powder. Yield: Eu**L** (72 mg, 95%), Gd**L** (16 mg, 21%), Tb**L** (74 mg, 80%), Nd**L** (78 mg, 85%), Sm**L** (74 mg, 79%), Dy**L** (78.7 mg, 77%), and Yb**L** (78 mg, 82%). Elemental analyses for Eu**L**: C_26_H_22_N_7_O_11_Eu (Calcd.: C 41.07; H 2.92; N 12.89. Found: C 41.1; H 2.95; N 12.76); Gd**L**: C_26_H_22_N_7_O_11_Gd (Calcd.: C 40.78; H 2.90; N 12.80. Found: C 40.73; H 2.92; N 12.62); Tb**L**: C_26_H_22_N_7_O_11_Tb (Calcd.: C 40.69; H 2.89; N 12.78. Found: C 40.74; H 2.94; N 12.65); Nd**L**: C_26_H_22_N_7_O_11_Nd (Calcd.: C 41.49; H 2.95; N 13.03. Found: C 41.54, H 2.92, N 12.89); Sm**L**: C_26_H_22_N_7_O_11_Sm (Calcd.: C 41.15, H 2.92, N 12.92. Found: C 41.09, H 2.86, N 12.82); Dy**L**: C_26_H_22_N_7_O_11_Dy (Calcd.: C 40.50, H 2.88, N 12.72. Found: C 40.46, H 2.86, N 12.42); and Yb**L**: C_26_H_22_N_7_O_11_Yb (Calcd.: C 39.96, H 2.84, N 12.55. Found: C 39.93, H 2.78, N 12.37).

### 4.4 Apparatus

Elemental analyses (C, H, and N) were performed using a vario EL (Elementar Analysensysteme GmbH Co.). ^1^H-NMR spectra were collected on a JEOL JNM-ECP 500. UV spectra and luminescence spectra were recorded on a Shimadzu UV-3600S and a Horiba Jobin Yvon Fluorolog 3-22, respectively. Absolute luminescence quantum yield values were measured using a Hamamatsu Photonics K.K. C9920-02 for the UV-vis wavelength region and C13534 for NIR. Structural analyses of a series of Ln**L** complexes were performed using a Rigaku Synergy S and XtaLAB mini II, Rigaku Oxford diffractometer with Mo K_*α*_ radiation (λ = 0.71073 Å). The structures were solved by direct methods and refined on *F* ^2^ by a full-matrix least-squares method using the SHELXTL-97 program: CCDC 2144933 (at 90 K) and 2144934 (at 300 K) for Eu**L**, 2144935 for Gd**L**, 2144936 for Tb**L**, 2144931 for Nd**L**, 2144932 for Sm**L**, 2144937 for Dy**L**, and 2144938 for Yb**L**. Synchrotron X-ray diffraction data were collected at the beam line BL02B2 (λ = 0.998983 Å) in SPring-8. The sample was held in a glass capillary (Markröhrchenaus Glas, 0.3 mm, Hilgenberg Co.)

## Data Availability

The datasets presented in this study can be found in online repositories. The names of the repository/repositories and accession number(s) can be found at: https://www.ccdc.cam.ac.uk/structures/—2144931—2144938.
