# Supplementary material for: Thermosensitive visible-light-excited visible-/NIR-luminescent complexes with lanthanide sensitized by the π-electronic system through intramolecular H-bonding
Source: Front Chem. 2022 Dec 7;10:1047960. doi: 10.3389/fchem.2022.1047960 (PMC9768490; doi:10.3389/fchem.2022.1047960)
Supplement: Supplementary file 1 [file DataSheet2.pdf]

Supplementary Material

Thermosensitive visible-light-excited visible-/NIR-luminescent complexes with a series of lanthanide ions sensitized by the π-electronic system through intramolecular H-bonding

Hitomi Ohmagari[a,b], Nicolas Marets[a,b], Jun Kamata[a], Mayo Yoneyama[a], Takumi Miyauchi[a], Yuta Takahashi[a], Yukina Yamamoto[a], Yuto Ogihara[a], Daisuke Saito[c,f], Kenta Goto[d], Ayumi Ishii[e], Masako Kato[f] and Miki Hasegawa[*a,b]

[a] *Department of Chemistry and Biological Science, Aoyama Gakuin University, 5-10-1 Fuchinobe, Chuo-ku, Sagamihara, Kanagawa 252-5258, Japan. E-mail: hasemiki@chem.aoyama.ac.jp; Fax: +81-42-759-6221*

[b] *Mirai Molecular Materials Design Institute, Aoyama Gakuin University, 5-10-1 Fuchinobe, Chuo-ku, Sagamihara, Kanagawa 252-5258, Japan*

[c] *Department of Chemistry, Faculty of Science, Hokkaido University, North-10 West-8, Kita-ku, Sapporo, Hokkaido 060-0810, Japan.*

[d] *Institute for Materials Chemistry and Engineering, Kyushu University, 744, Motooka, Nishi-ku, Fukuoka, 819-0395, Japan.*

[e] *Department of Natural & Environmental Science, Teikyo University of Science, 2525 Yatsuzawa, Uenohara, Yamanashi 409-0193, Japan.*

[f] *Department of Applied Chemistry for Environment, School of Biological and Environmental Sciences, Kwansei Gakuin University, 1 Gakuen Uegahara, Sanda, Hyogo 669-1330, Japan.*

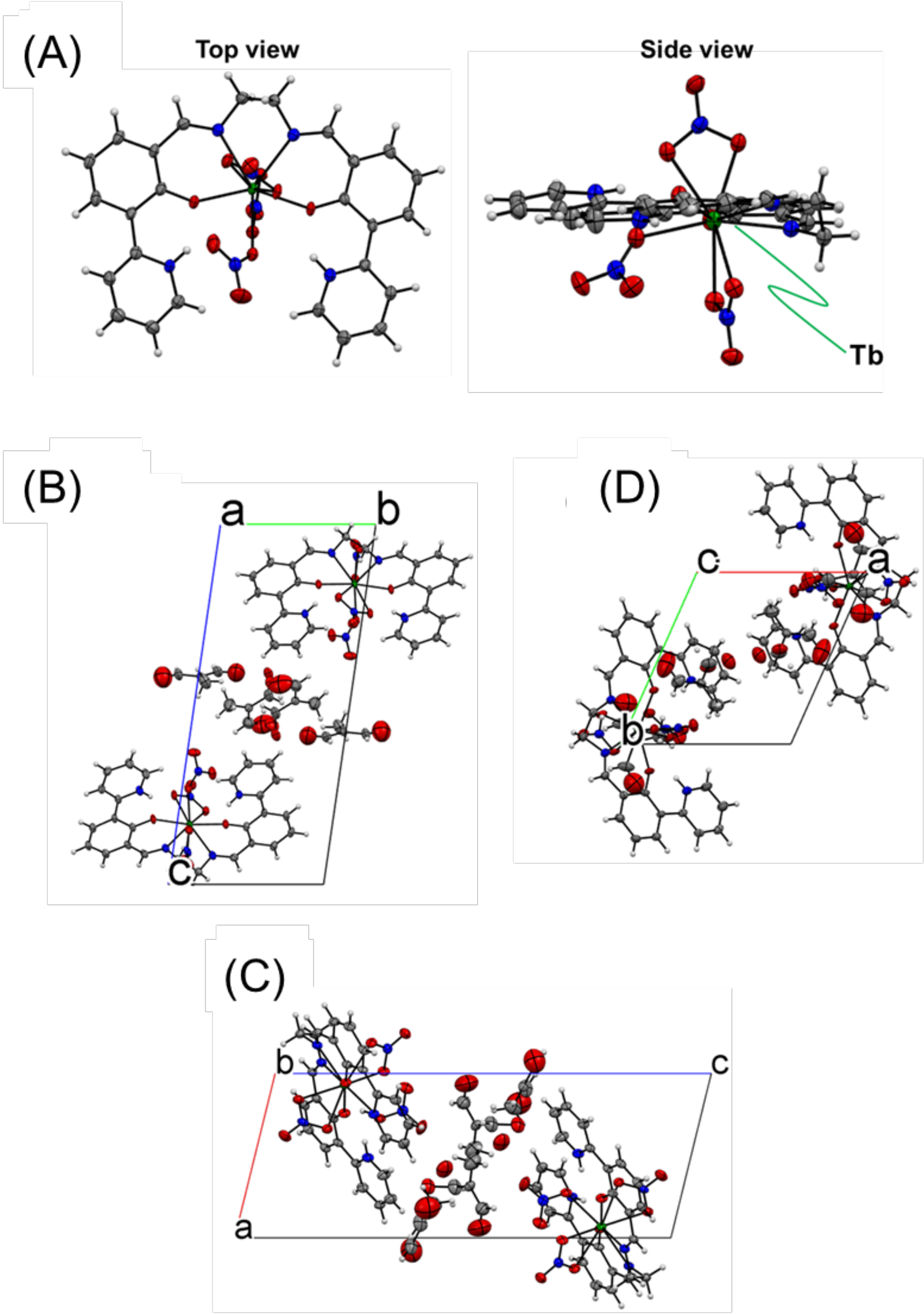

Figure S1. (A) ORTEP drawings and molecular packing of Tb**L** projected from (B) *a*-axis, (C) *b*-axis and (D) *c*-axis observed at 90 K. Gray, white, red, blue and green are C, H, O, N and Tb, respectively.

Table S1. Crystallographic data of Eu**L** observed at 90 and 300 K.

	at 90 K (CCDC: 2144933)	at 300K (CCDC: 2144934)
Formula	$C_{26}H_{22}N_7O_{11}Eu$	$C_{26}H_{22}N_7O_{11}Eu$
Formula weight	760.47	760.46
Crystal size (mm)	0.181×0.093×0.049	0.304×0.165×0.135
Crystal system	Triclinic	Triclinic
Space group	*P*-1	*P*-1
a (Å)	9.4025(2)	9.4102(7)
b (Å)	10.0770(3)	10.1547(7)
c (Å)	16.1076(5)	16.4151(8)
α (°)	79.693(3)	79.409(5)
β (°)	82.996(2)	82.490(6)
γ (°)	66.283(3)	66.174(5)
V(Å^3)	1372.62(7)	1407.68(18)
Z value	2	2
D_{calcd} (Mg m^{-3})	1.840	1.794
μ (Cu Kα) (mm^{-1})	17.034	—
μ (Mo Kα) (mm^{-1})	—	2.303
F (000)	756	756
λ (Cu Kα) (Å)	1.54184	—
λ (Mo Kα) (Å)	—	0.71073
Temp. (K)	90	300
$R_1{}^a$ ($I > 2.00\sigma(I)$)	0.0275	0.0645
w$R_2{}^b$ ($I > 2.00\sigma(I)$)	0.0725	0.1586
Goodness of fit	1.054	1.052
Largest peak and hole (e Å^{-3})	0.591	1.38
Apparatus	Rigaku XtaLAB Synergy-S	Rigaku XtaLAB Mini II

^{a} $R_1 = \Sigma||F_o| - |F_c||/\Sigma|F_o|$. ^{b} w$R_2 = \{\Sigma[w(F_o{}^2 - F_c{}^2)^2]/\Sigma[w(F_o{}^2)^2]\}^{1/2}$.

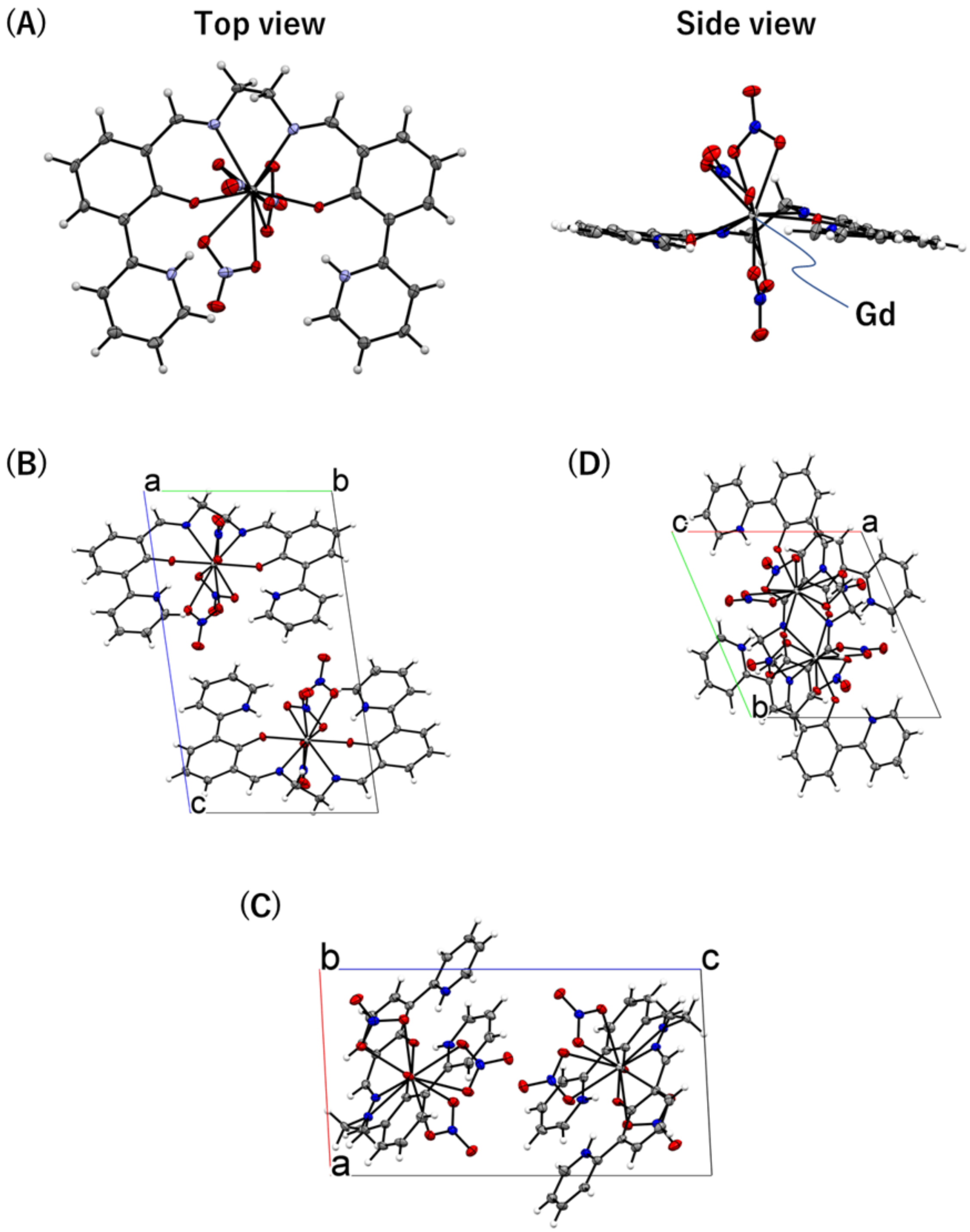

Figure S2. (A) ORTEP drawings and molecular packing of Gd**L** projected from (B) *a*-axis, (C) *b*-axis and (D) *c*-axis observed at 90 K. Gray, white, red, blue and pale grey are C, H, O, N and , respectively.

Table S2. Crystallographic data of Tb**L** and Gd**L** at 90 K.

	Tb**L**	Gd**L**
Formula	$C_{26}H_{22}N_7O_{11}Tb$	$C_{26}\ H_{22}N_7O_{11}Gd$
Formula weight	937.58	765.75
Crystal size (mm)	0.302×0.124×0.083	0.257×0.109×0.03
Crystal system	Triclinic	Triclinic
Space group	*P*-1	*P*-1
a (Å)	9.1926(2)	9.3942(6)
b (Å)	9.9413(2)	10.0769(5)
c (Å)	21.8688(3)	16.0808(7)
α (°)	93.0080(10)	79.855(4)
β (°)	101.5280(10)	83.059(4)
γ (°)	112.809(2)	66.327(5)
V(Å^3)	1786.29(6)	1373.1(2)
Z value	2	2
D_{calcd} (Mg m^{-3})	1.746	1.852
μ (Cu Kα) (mm^{-1})	10.386	-
μ (Mo Kα) (mm^{-1})	-	2.492
F (000)	940	758
λ(Cu Kα) (mm^{-1})	1.54184	-
λ (Mo Kα) (Å)	-	-0.71073
Temp. (K)	90	100
$R_1{}^a$ ($I > 2.00\sigma(I)$)	0.0646	0.0353
w$R_2{}^b$ ($I > 2.00\sigma(I)$)	0.1859	0.0953
Goodness of fit	1.0957	1.111
Largest peak and hole (e Å^{-3})	.612, -2.038	0.69, -1.46
Apparatus	Rigaku XtaLAB Synergy-S	Rigaku XtaLAB Mini II

^{a} $R_1 = \Sigma||F_o| - |F_c||/\Sigma|F_o|$. ^{b} w$R_2 = \{\Sigma[w(F_o{}^2 - F_c{}^2)^2]/\Sigma[w(F_o{}^2)^2]\}^{1/2}$.

Table S3. Selected bond lengths (Å) of Ln**L**.

	Nd**L**	Sm**L**	Eu**L**	Gd**L**	Tb**L**	Dy**L**	Yb**L**
Ln-O(3)	2.59	2.59	2.56	2.54	2.49	2.47	2.42
Ln-O(4)	2.61	2.59	2.57	2.65	2.54	2.54	2.53
Ln-O(6)	2.57	2.56	2.65	2.54	2.47	2.45	2.40
Ln-O(7)	2.62	2.65	2.55	2.65	3.30	—	—
Ln-O(9)	2.54	2.53	2.51	2.49	2.45	2.44	2.40
Ln-O(10)	2.60	2.62	2.60	2.59	2.55	2.54	2.51

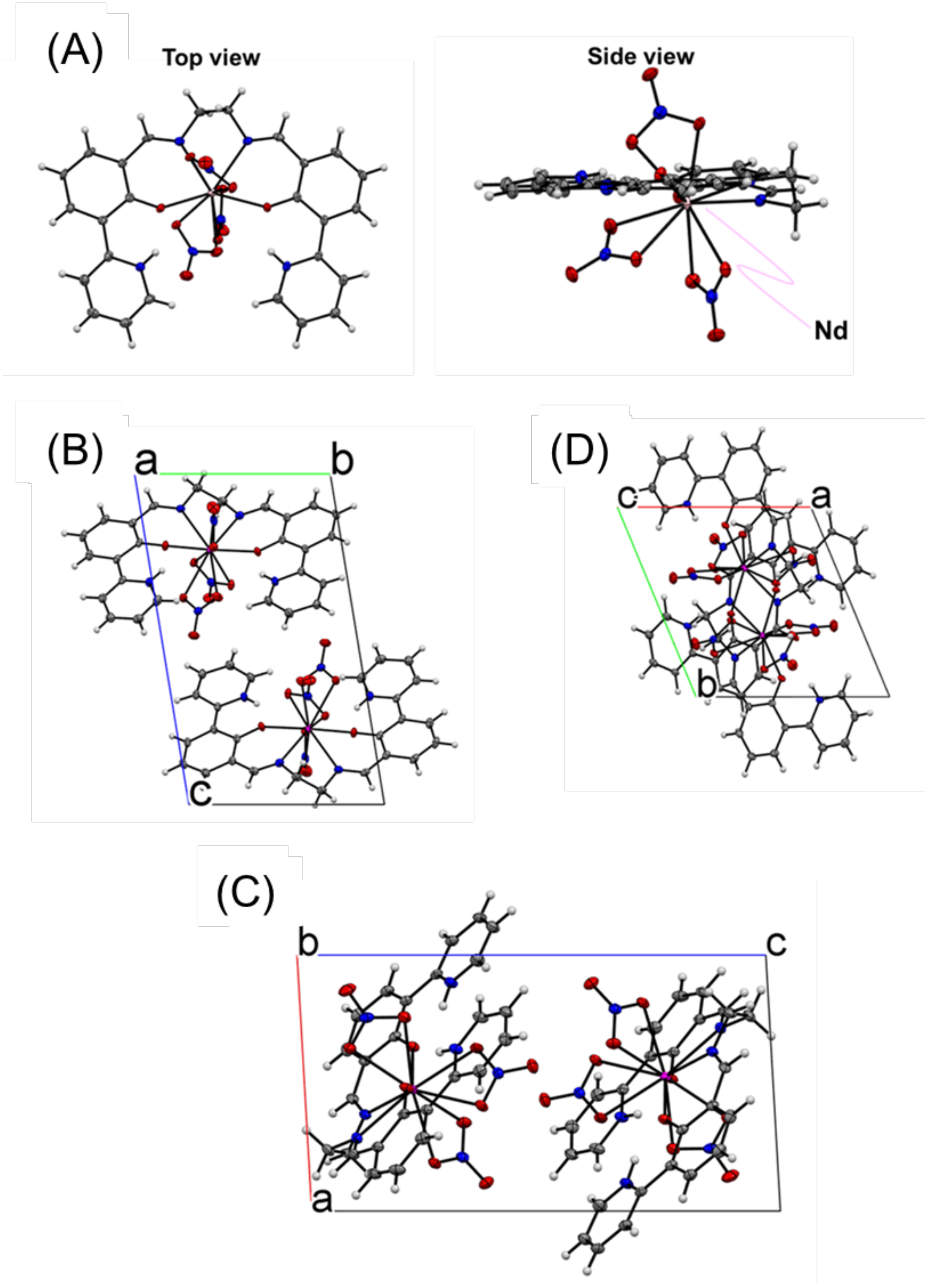

Figure S3. (A) ORTEP drawings and molecular packing of Nd**L** projected from (B) *a*-axis, (C) *b*-axis and (D) *c*-axis observed at 77 K. Gray, white, red, blue and violet are C, H, O, N and Nd, respectively.

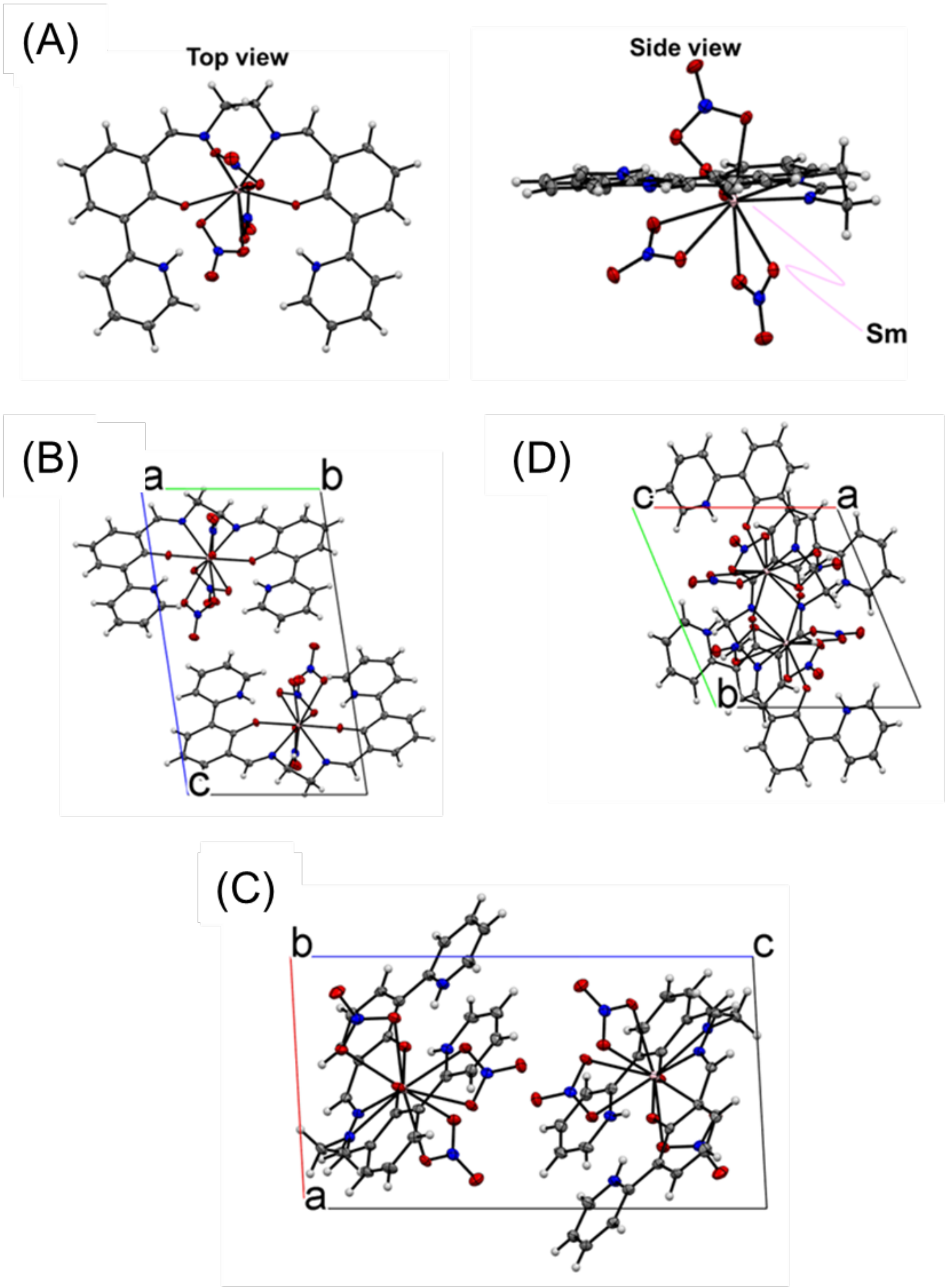

Figure S4. (A) ORTEP drawings and molecular packing of Sm**L** projected from (B) *a*-axis, (C) *b*-axis and (D) *c*-axis observed at 77 K. Gray, white, red, blue and pink are C, H, O, N and Sm, respectively.

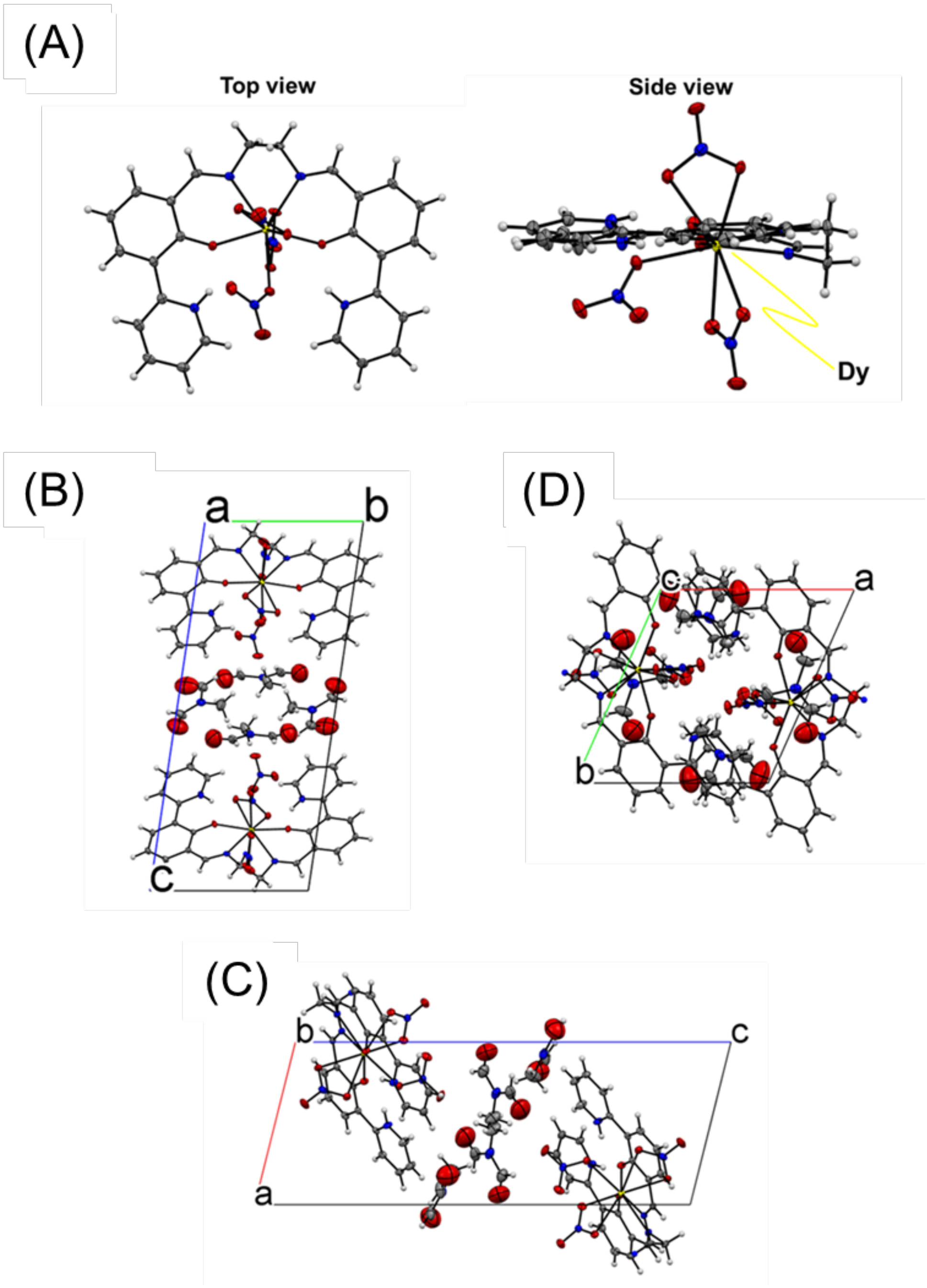

Figure S5. (A) ORTEP drawings and molecular packing of Dy**L** projected from (B) *a*-axis, (C) *b*-axis and (D) *c*-axis observed at 77 K. Gray, white, red, blue and yellow are C, H, O, N and Tb, respectively.

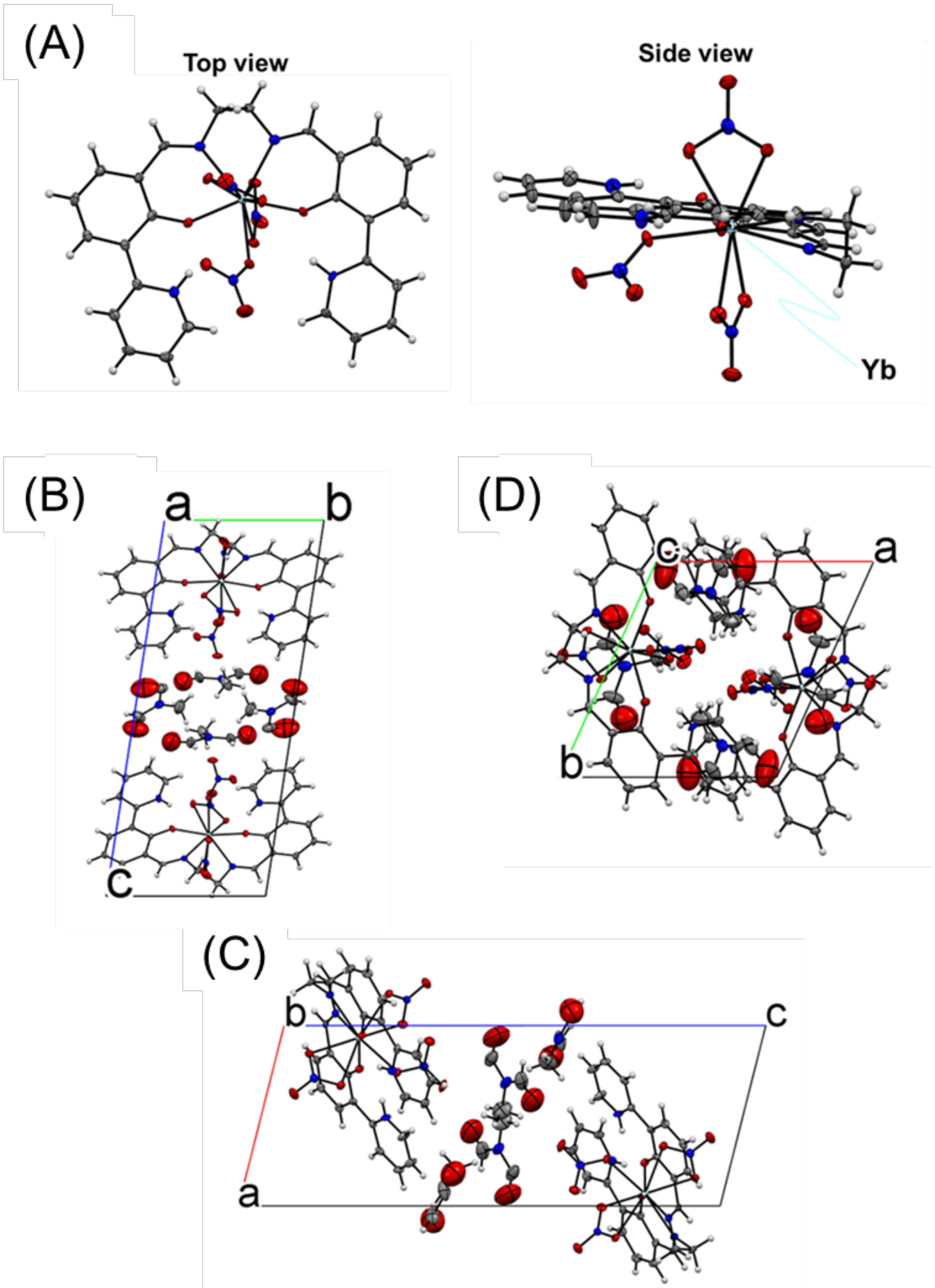

Figure S6. (A) ORTEP drawings and molecular packing of Yb**L** projected from (B) *a*-axis, (C) *b*-axis and (D) *c*-axis observed at 77 K. Gray, white, red, blue and sky-blue are C, H, O, N and Tb, respectively.

Table S4. Crystallographic data of Nd**L**, Sm**L**, Dy**L** and Yb**L**.

	Nd**L**	Sm**L**	Dy**L**	Yb**L**
Formula	$C_{26}H_{22}N_7O_{11}Nd$	$C_{26}H_{22}N_7O_{11}Sm$	$C_{32}H_{31}N_9O_{15}Dy$	$C_{32}H_{31}N_9O_{15}Yb$
Formula weight	752.74	758.85	943.96	954.50
Crystal system	Triclinic	Triclinic	Triclinic	Triclinic
Space group	*P*-1	*P*-1	*P*-1	*P*-1
a (Å)	9.3785(3)	9.4436(5)	9.1740(3)	9.2213(4)
b (Å)	10.0769(3)	10.1057(4)	9.8613(4)	9.8213(4)
c (Å)	16.0187(4)	16.1319(7)	21.9422(4)	22.0335(7)
α (°)	78.537	79.171	93.208	93.438
β (°)	82.520	82.811	101.620	101.691
γ (°)	66.603	66.359	112..643	112.890
V(Å^3)	1359.46(7)	1383.09(12)	1774.83(7)	1776.83
Z value	2	2	2	2
D_{calcd} (Mg m^{-3})	1.839	1.822	1.769	1.784
μ (Mo Kα) (mm^{-1})	1.987	2.199	2.192	2.718
F (000)	750	754	946	952
λ (Mo Kα) (Å)	0.71073	0.71073	0.71073	0.71073
Temp. (K)	100	100	100	100
$R_1{}^a$ (I >2.00$\sigma(I)$)	0.0266	0.0398	0.0327	0.0468
w$R_2{}^b$ (I >2.00$\sigma(I)$)	0.0592	0.0749	0.0849	0.1085
Goodness of fit	1.029	0.997	1.077	1.031
Largest peak and hole (e Å^{-3})	0.695, -1.068	1.284, -0.775	1.890, -0.969	2.190, -1.246
Apparatus	Rigaku XtaLAB Mini II	Rigaku XtaLAB Mini II	Rigaku XtaLAB Mini II	Rigaku XtaLAB Mini II

^{a} $R_1 = \Sigma||F_o| - |F_c||/\Sigma|F_o|$. ^{b} w$R_2 = \{\Sigma[w(F_o{}^2 - F_c{}^2)^2]/\Sigma[w(F_o{}^2)^2]\}^{1/2}$.

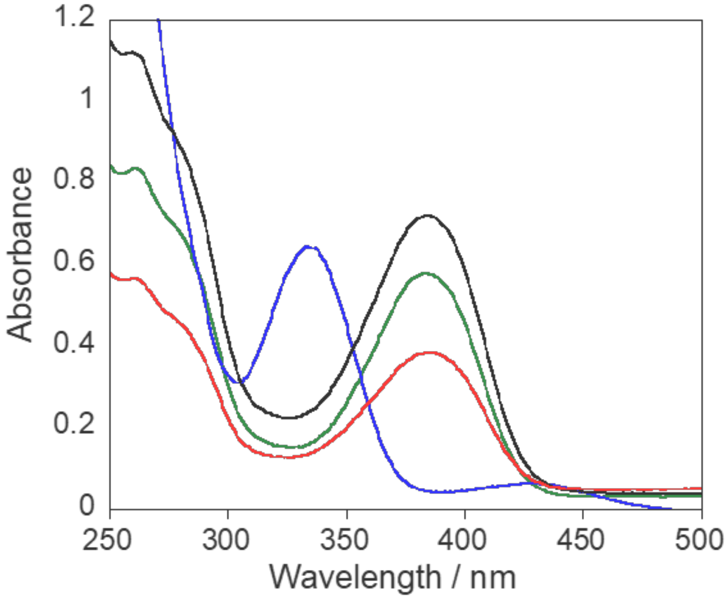

Figure S7. Electronic absorption spectra of **L** (blue), Eu**L** (red), Tb**L** (green) and Gd**L** (black) in acetonitrile.

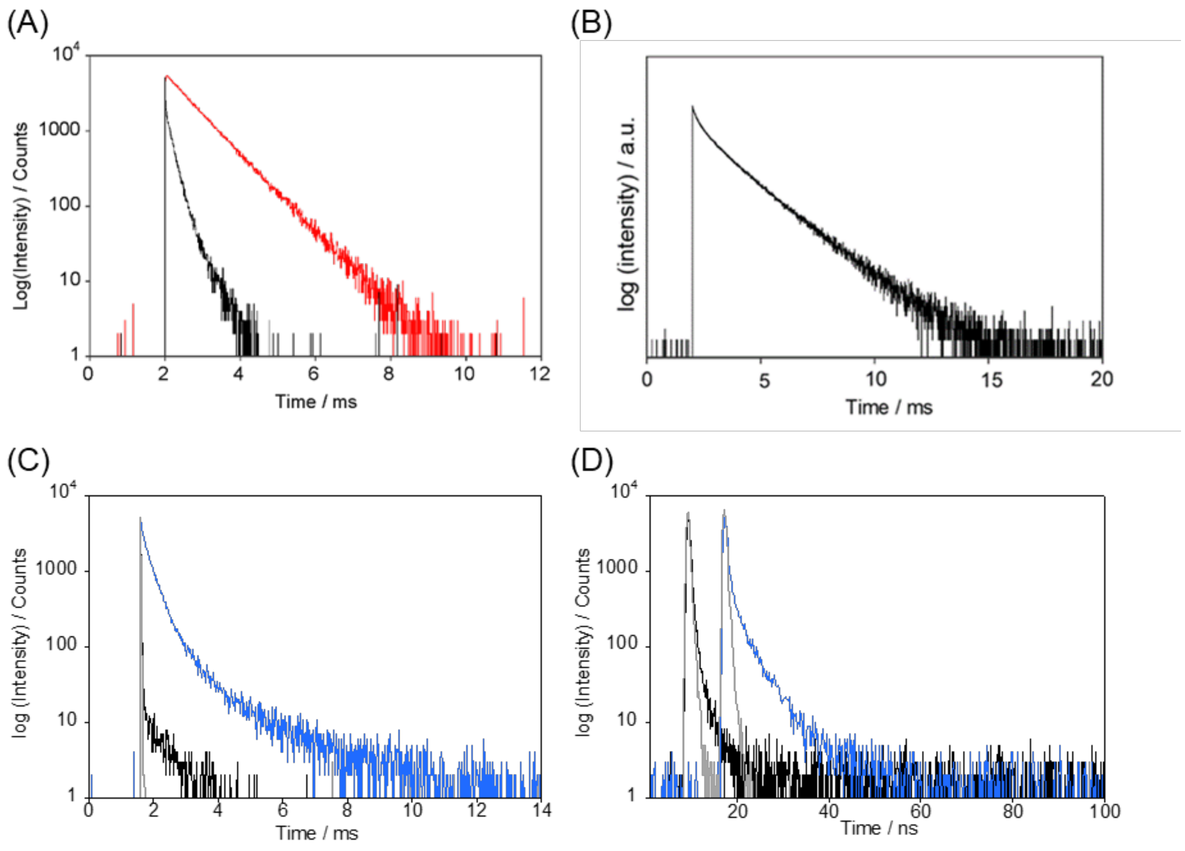

Figure S8. Luminescence decay curves of (A) Eu**L** (black: at rt, red: at 77 K), λ_{ex} = 365 nm, λ_{mon} = 621 nm), (B) Tb**L** (black: at 77 K, λ_{ex} = 370 nm, λ_{mon} = 542 nm) and (C, D) Gd**L** (black: at rt, blue: at 77 K), (C) λ_{ex} = 370 nm, λ_{mon} = 540 nm, (D) λ_{ex} = 365 nm, λ_{mon} = 470 nm,) in the solid state.

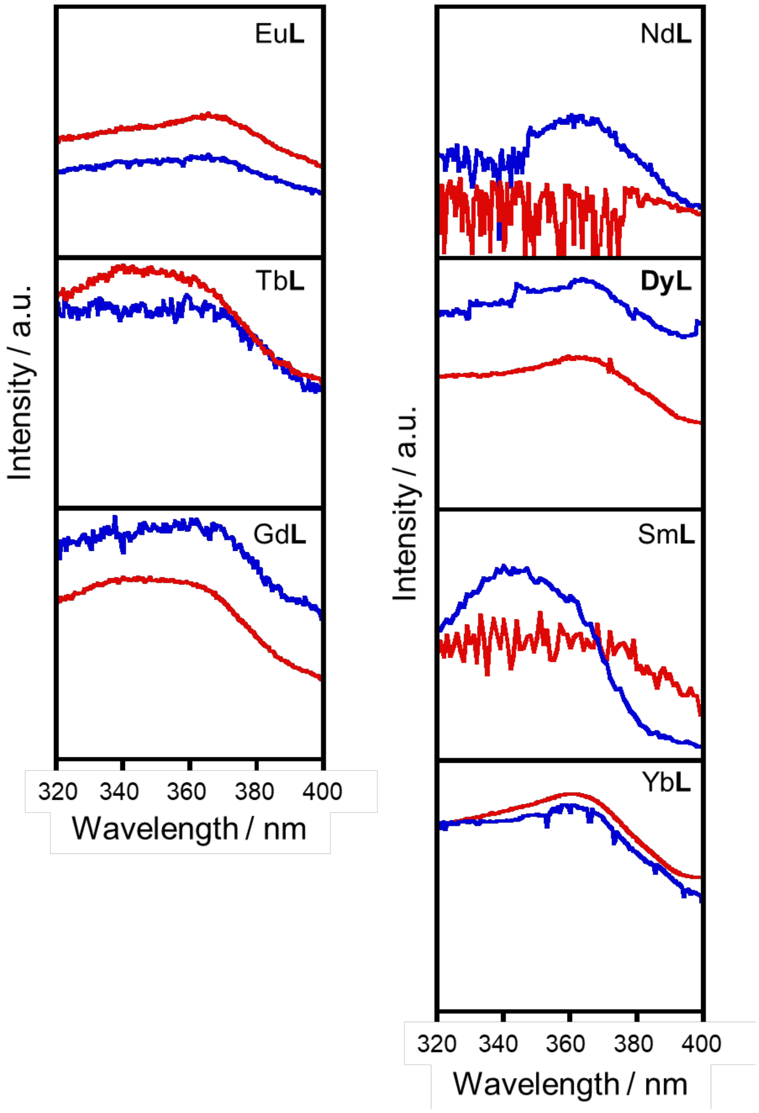

Figure S9. Excitation spectra of Ln**L** (Ln = Eu, Tb, Gd, Nd, Dy, Sm and Yb) at rt(red) and 77 K(blue) in the solid state. Monitor wavelengths were 621, 542, 530, 1074, 952, 1113 and 979 nm for Eu**L**, Tb**L**, Gd**L**, Nd**L**, Dy**L**, Sm**L** and Yb**L**, respectively.

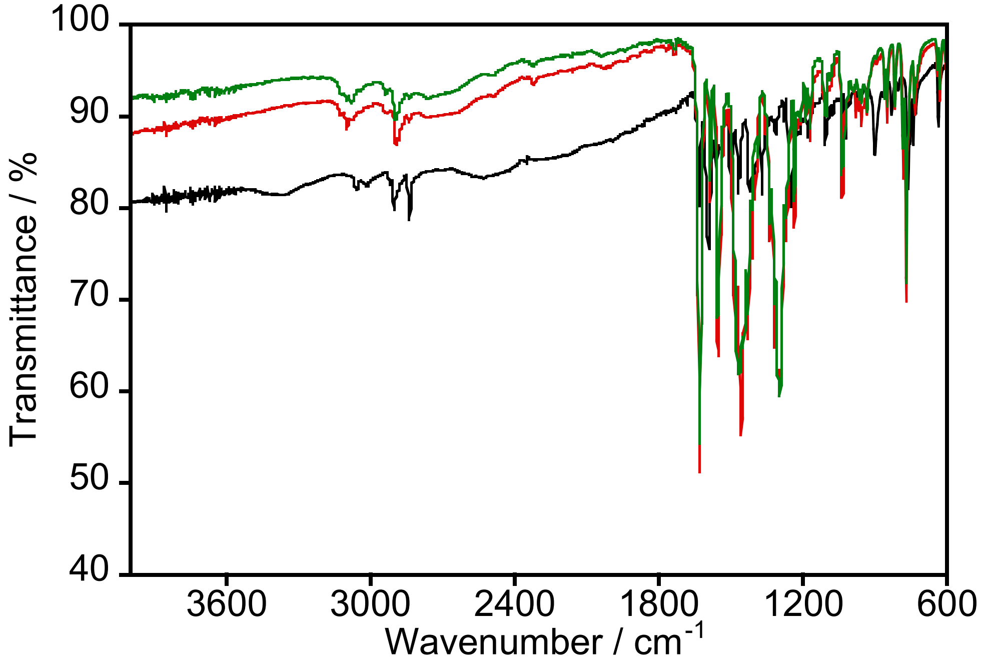

Figure S10. FT-IR spectra of Eu**L**(red), Tb**L**(green) and their ligand **L**(black) observed with ATR system (Thermo Fisher Scientific, Nicolet iS50).
